# Selection strategy of dextran sulfate sodium-induced acute or chronic colitis mouse models based on gut microbial profile

**DOI:** 10.1186/s12866-021-02342-8

**Published:** 2021-10-16

**Authors:** Hao-Ming Xu, Hong-Li Huang, Yan-Di Liu, Jia-Qi Zhu, You-Lian Zhou, Hui-Ting Chen, Jing Xu, Hai-Lan Zhao, Xue Guo, Wei Shi, Yu-Qiang Nie, Yong-Jian Zhou

**Affiliations:** 1 Department of Gastroenterology and Hepatology, Guangzhou Digestive Disease Center, Guangzhou First People’s Hospital, School of Medicine, South China University of Technology, No. 1 Panfu Road, Guangzhou, 510180 China; 2 Department of Geriatrics, Guangzhou First People’s Hospital, School of Medicine, South China University of Technology, Guangzhou, 510180 China

**Keywords:** Dextran sulfate sodium, Acute colitis, Chronic colitis, Gut microbiota

## Abstract

**Background:**

Dextran sulfate sodium (DSS) replicates ulcerative colitis (UC)-like colitis in murine models. However, the microbial characteristics of DSS-triggered colitis require further clarification. To analyze the changes in gut microbiota associated with DSS-induced acute and chronic colitis.

**Methods:**

Acute colitis was induced in mice by administering 3% DSS for 1 week in the drinking water, and chronic colitis was induced by supplementing drinking water with 2.5% DSS every other week for 5 weeks. Control groups received the same drinking water without DSS supplementation. The histopathological score and length of the colons, and disease activity index (DAI) were evaluated to confirm the presence of experimental colitis. Intestinal microbiota was profiled by *16S rDNA* sequencing of cecal content.

**Results:**

Mice with both acute and chronic DSS-triggered colitis had significantly higher DAI and colon histopathological scores in contrast to the control groups (*P* < 0.0001, *P* < 0.0001), and the colon was remarkably shortened (*P* < 0.0001, *P* < 0.0001). The gut microbiota α-diversity was partly downregulated in both acute and chronic colitis groups in contrast to their respective control groups (Pielou index *P* = 0.0022, *P* = 0.0649; Shannon index *P* = 0.0022, *P* = 0.0931). The reduction in the Pielou and Shannon indices were more obvious in mice with acute colitis (*P* = 0.0022, *P* = 0.0043). The relative abundance of *Bacteroides* and *Turicibacter* was increased (all *P* < 0.05), while that of *Lachnospiraceae*, *Ruminococcaceae*, *Ruminiclostridium*, *Rikenella*, *Alistipes*, *Alloprevotella,* and *Butyricicoccus* was significantly decreased after acute DSS induction (all *P* < 0.05). The relative abundance of *Bacteroides*, *Akkermansia*, *Helicobacter*, *Parabacteroides*, *Erysipelatoclostridium*, *Turicibacter* and *Romboutsia* was also markedly increased (all *P* < 0.05), and that of *Lachnospiraceae_NK4A136_group*, *Alistipes*, *Enterorhabdus*, *Prevotellaceae_UCG-001*, *Butyricicoccus*, *Ruminiclostridium_6*, *Muribaculum*, *Ruminococcaceae_NK4A214_group*, *Family_XIII_UCG-001* and *Flavonifractor* was significantly decreased after chronic DSS induction (all *P* < 0.05).

**Conclusion:**

DSS-induced acute and chronic colitis demonstrated similar symptoms and histopathological changes. The changes in the gut microbiota of the acute colitis model were closer to that observed in UC. The acute colitis model had greater abundance of SCFAs-producing bacteria and lower α-diversity compared to the chronic colitis model.

**Supplementary Information:**

The online version contains supplementary material available at 10.1186/s12866-021-02342-8.

## Background

Inflammatory bowel disease (IBD) comprises of Crohn’s disease (CD) and ulcerative colitis (UC). The hallmark of CD is chronic colorectal inflammation, which manifests as diarrhea, bloody mucus stool, abdominal pain and other symptoms that severely affect patients’ quality of life. The pathogenesis of UC is complex, and involves genetic, immunological and microbiological factors.

The dextran sodium sulfate (DSS)-stimulated colitis model mimics the pathological damage and symptoms of human UC [1], and is routinely used for studying the pathogenesis and pharmacodynamics of UC [2]. DSS is a water-soluble negatively-charged sulfated polysaccharide that can erode the intestinal mucosa [3, 4]. DSS increases colon permeability by destroying interepithelial cell tight junctions, reducing mucin levels while simultaneously altering the resident microbiota [5, 6]. A compromised intestinal barrier allows penetration of harmful bacterial from the intestinal lumen into the systemic circulation, thereby triggering an inflammatory response. The efficacy of colitis induction depends on the concentration of DSS (typically 1–5%), dosage regimen (long- or short-term), animal strains (BALB/c and C57BL/6 mice are more susceptible), and feeding conditions [7]. With the development of *16S rDNA* sequencing, the DSS-induced colitis model been proven to be particularly well-suited for analyzing colitis-associated microbial changes. DSS-induced acute and chronic colitis mimic histopathological damages associated with UC. Intermittent administration of different concentrations of DSS over varying durations result in alternating active and remission phases, which is reflective of disease progression in UC patients. In order to establish acute and chronic models of colitis in mice, either 3% or 2.5% DSS is added to the drinking water for varying durations [8, 9].

Short chain fatty acids (SCFAs) are organic fatty acids with aliphatic tails of six or fewer carbons, including acetate, propionate, butyrate, valproate and hexanoate, that are mainly produced by anaerobic microorganisms while fermenting indigestible carbohydrates. SCFAs inhibit the production of inflammatory cytokines in human intestinal epithelial cells in vitro, which promotes intestinal mucosal repair by mitigating inflammation [10]. In vitro studies have shown that SCFAs also exert an anti-tumor effect by reducing the secretion of tumor necrosis factor in intestinal epithelial cells [11]. In addition, SCFAs (especially butyrate) regulate gut microbiota by modulating the intestinal lumen pH to levels that are conducive to the growth of SCFAs-producing bacteria [12]. Acetate, propionate and butyrate are the most abundant SCFAs produced during anaerobic fermentation of dietary fibers in the intestine, and account for more than 85% of all SCFAs. The relative proportion of the three is about 3:1:1 [13], and affected by the host genotype, intestinal microbial composition and diet. Anaerobic bacteria in the colon play an important role in fermentation. The main SCFAs producing-bacteria in the human gut belong to the phylum Firmicutes, in particular *Faecalibacterium prausnitzii* and *Clostridium leptum* of family *Ruminococcaceae*, *Eubacterium rectale* and *Roseburia spp.* of *Lachnospiraceae*, as well as *Butyricicoccus*, *Rikenella* and *Alistipes* [14, 15]. In addition, the mucin-degrading bacteria *Akkermansia muciniphila* (phylum *Verrucomicrobia*) produces both propionate and acetate [16].

Consistent with our previous studies [17–20], we found that DSS-triggered acute and chronic colitis were similar in terms of disease activity, colon shortening and histopathological changes in the colon, but differed remarkably in terms of gut microbiota profiles. Given that the initiation and progression of UC centers heavily on gut microbiota proportions, the therapeutic potential of fecal microbiota transplantation is increasingly being assessed in UC animal models. The selection of appropriate colitis mouse models vis-à-vis the gut microflora has become particularly important. In this study, we analyzed the changes in gut microbiota associated with DSS-triggered acute and chronic colitis in a mouse model in order to provide a reference basis for selecting an appropriate model.

## Results

### Acute and chronic colitis were successfully induced by DSS exposure

**Fig. 1:**
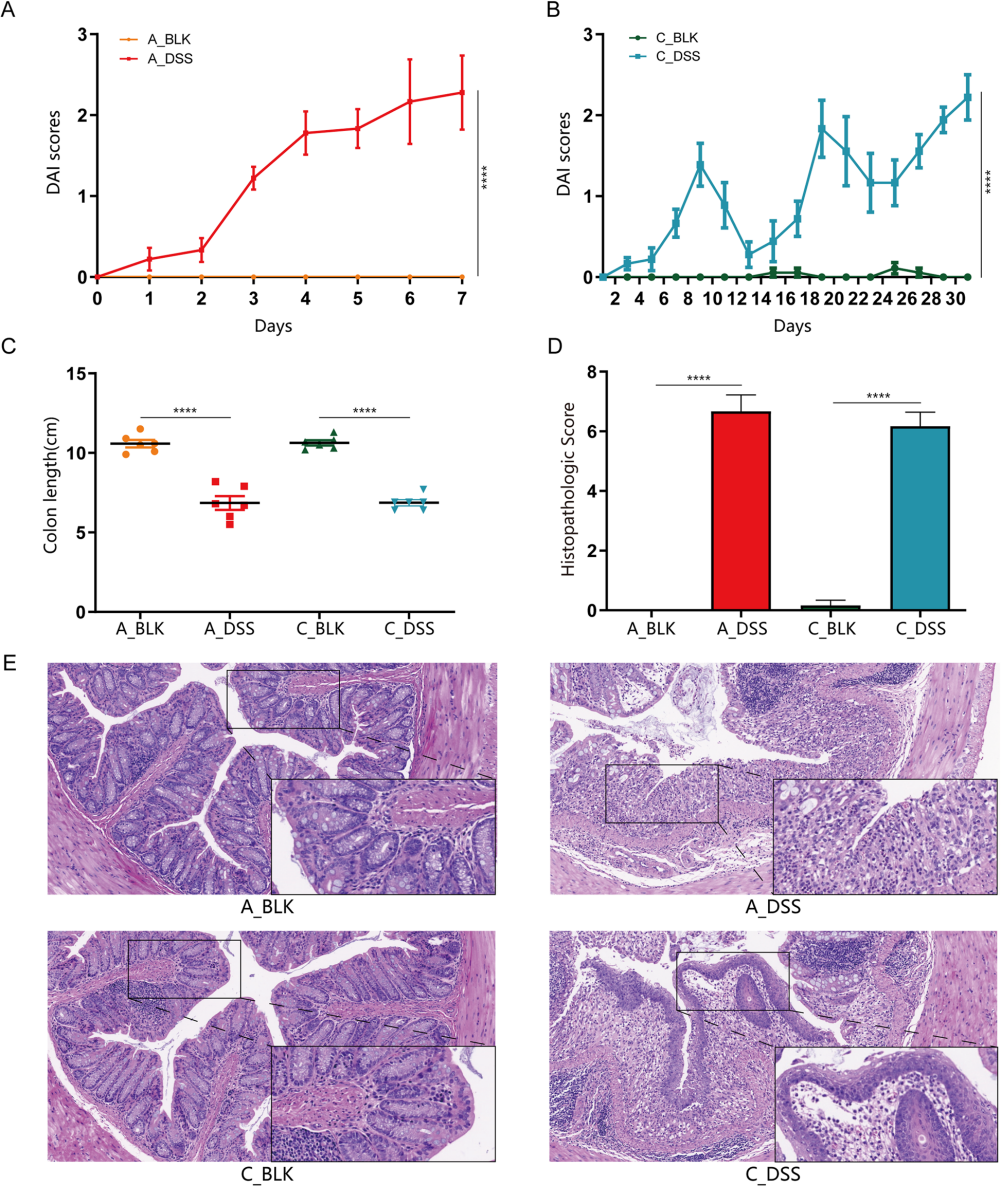
DSS significantly induced inflammation in the colons of mice. **A, B** Disease activity index (DAI) dynamics across each cohort. **C** Mouse colon lengths of each group. **D** Colon histopathologic score of each group of mice. **E** Colonic tissues stained with H&E (200x; 400x). Data is depicted in terms of individual means or the mean ± SD of each group derived from three experimental replicates. ****P < 0.0001

Mice exposed to DSS of varying concentrations and doses successfully developed acute and chronic colitis [8]. Based on preliminary results, we administered 3% DSS for 1 week to stimulate acute colitis and 2.5% DSS intermittently for 5 weeks to induce chronic colitis. As shown in Fig. 1A and B, the DAI scores of mice increased significantly after 1 week of administering 3% DSS compared to the untreated controls (*P* < 0.0001). However, the DAI scores fluctuated slightly after 14 days during chronic colitis modeling, corresponding to transient weight loss without change in stool characteristics. This can be attributed to intermittent DSS administration over the 5-week period. Nevertheless, DAI scores were notably raised in the chronic colitis group relative to the control group (*P* < 0.0001) after 5 weeks. Both acute and chronic colitis groups demonstrated no remarkable differences in terms of DAI scores (*P* = 0.9198). As shown in Fig. 1C**,** both forms of colitis were accompanied by significant shortening of the colon compared to their respective controls (*P* < 0.0001 for both), and the colon lengths did not vary significantly between the acute and chronic colitis models (*P* = 0.9724). Histopathological examination of the colon tissues of untreated control mice indicated clearly demarcated mucosa, intact epithelium, neatly arranged glands, abundant goblet cells, and absence of inflammatory cell infiltrates in the lamnia propria. In contrast, the DSS-treated mice exhibited considerable structural damage including incomplete glands, loss of epithelial cells, as well as extensive inflammatory cell infiltrates. Interestingly, the acute colitis group demonstrated notably thinned intestinal mucosa in contrast to that seen in the chronic colitis group. Mice with chronic colitis demonstrated locally thickened mucosa, which could be the result of alternating inflammatory and remission phases during the modeling process (Fig. 1E). Nevertheless, the histopathological scores of both groups were similar (*P* = 0.5113), and markedly higher compared to that of the respective controls (*P* < 0.0001 for both) (Fig. 1D).

### Acute and chronic DSS exposure distinctly altered the diversity of the intestinal microflora

**Fig. 2:**
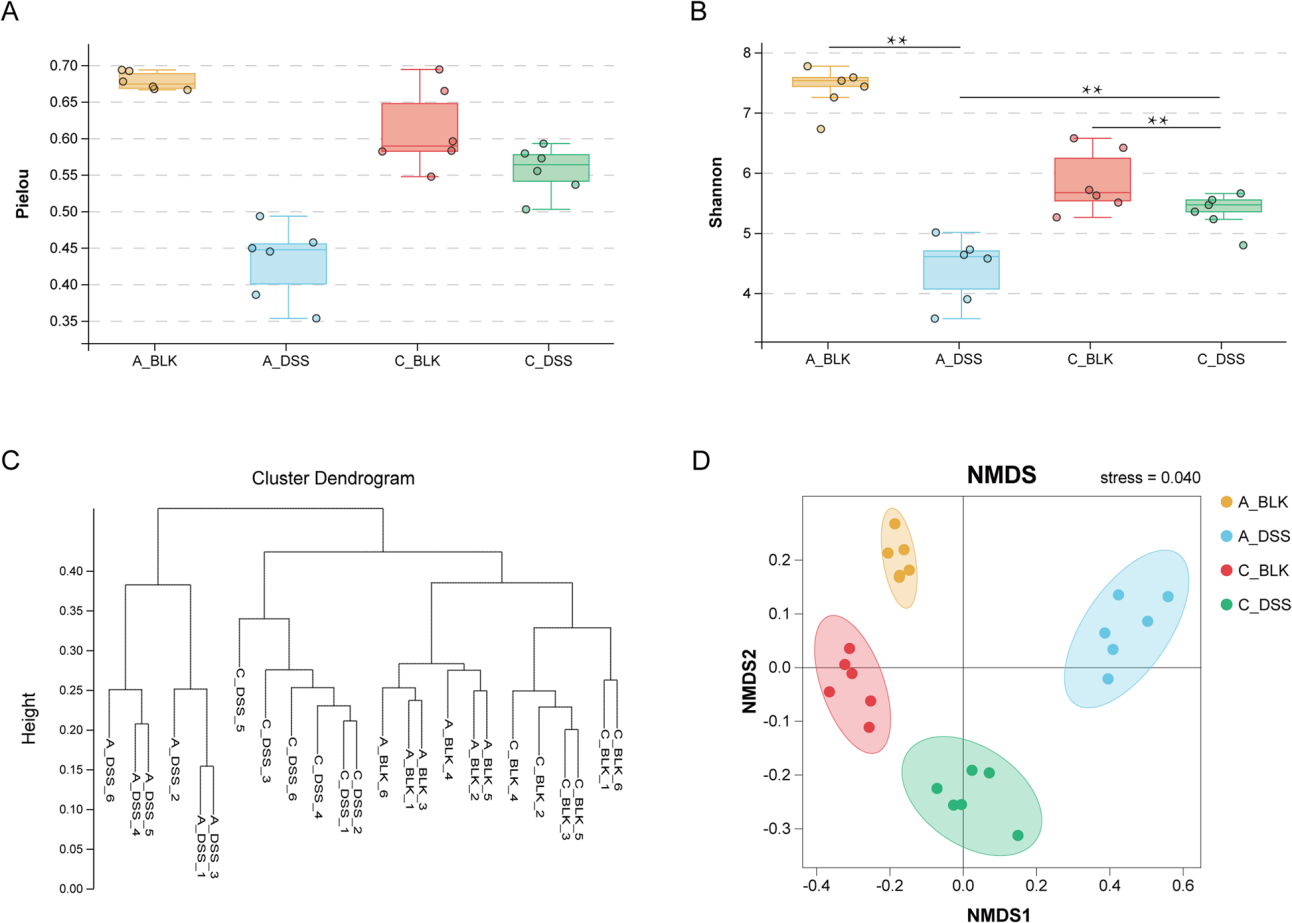
DSS alters diversity of gut microbiota in mice models of colitis. **A** Pielou index. **B** Shannon index. α-diversity was derived using a combination of the species richness (species situation) and species evenness (distribution) using the Pielou and Shannon indices. **C** Gut microbiota relationships and distribution are characterized with a UPGMA clustering tree. Samples are represented by end branches. In general, samples belonging to similar groups were clustered into a large branch with different branches representing different groups. Distance is indicated by the vertical axis. Samples with similar structure of bacterial communities are located in the same branch. **D** NMDS (Non-metric Multi-Dimensional Scaling). NMDS representas a dimension reduction analysis established on distance ranking. It is superior to linear models (including PCA and PCoA) and more accurately translates the nonlinear structure of ecological data. The accuracy of the model is evaluated by the stress value. The smaller the stress value is, the more reliable the model is. Generally, the stress value less than 0.1 is better. **P < 0.01

The intestinal microbiota profiles were analyzed by *16S rDNA* sequencing. The Pielou index (Fig. 2A), which is indicative of species evenness, was notably lower in both the acute (*P* = 0.0022) and chronic (*P* = 0.0649) colitis groups compared to the respective controls, and did not differ significantly between the forms of colitis (*P* = 0.0022). The Shannon index (Fig. 2B) is a measure of species richness and evenness, and was decreased in both acute colitis (*P* = 0.0022) and chronic colitis (*P* = 0.0931) groups in contrast to the respective controls. Furthermore, acute colitis resulted in significantly lower Shannon index compared to chronic colitis (*P* = 0.0043). Taken together, DSS-induced colitis lowers mice gut microbiota species diversity, with more drastic changes seen in models of acute colitis.

The β-diversities of the intestinal microbiota across both cohorts were assessed using UPGMA and FastTree (Fig. 2C), NMDS (Fig. 2D), PCA (Fig. S1A), and PCoA (Fig. S1B). The results of UPGMA and FastTree showed that the acute colitis and control groups were further apart compared to the chronic colitis group and its corresponding control, indicating that acute colitis induced more significant changes in the intestinal microflora. The distance between acute and chronic colitis mice models was halfway between each colitis cohort and its respective control. Consistent results were obtained with NMDS, PCA and PCoA (stress< 0.1, PCA1 + PCA2 > 50%, PCoA1 + PCoA2 > 50%). The acute colitis group was the farthest from the rest, indicating that acute exposure to DSS induced significant changes in the gut microbial structure.

### Acute and chronic DSS-triggered colitis resulted in a distinct microbiota profile

**Fig. 3:**
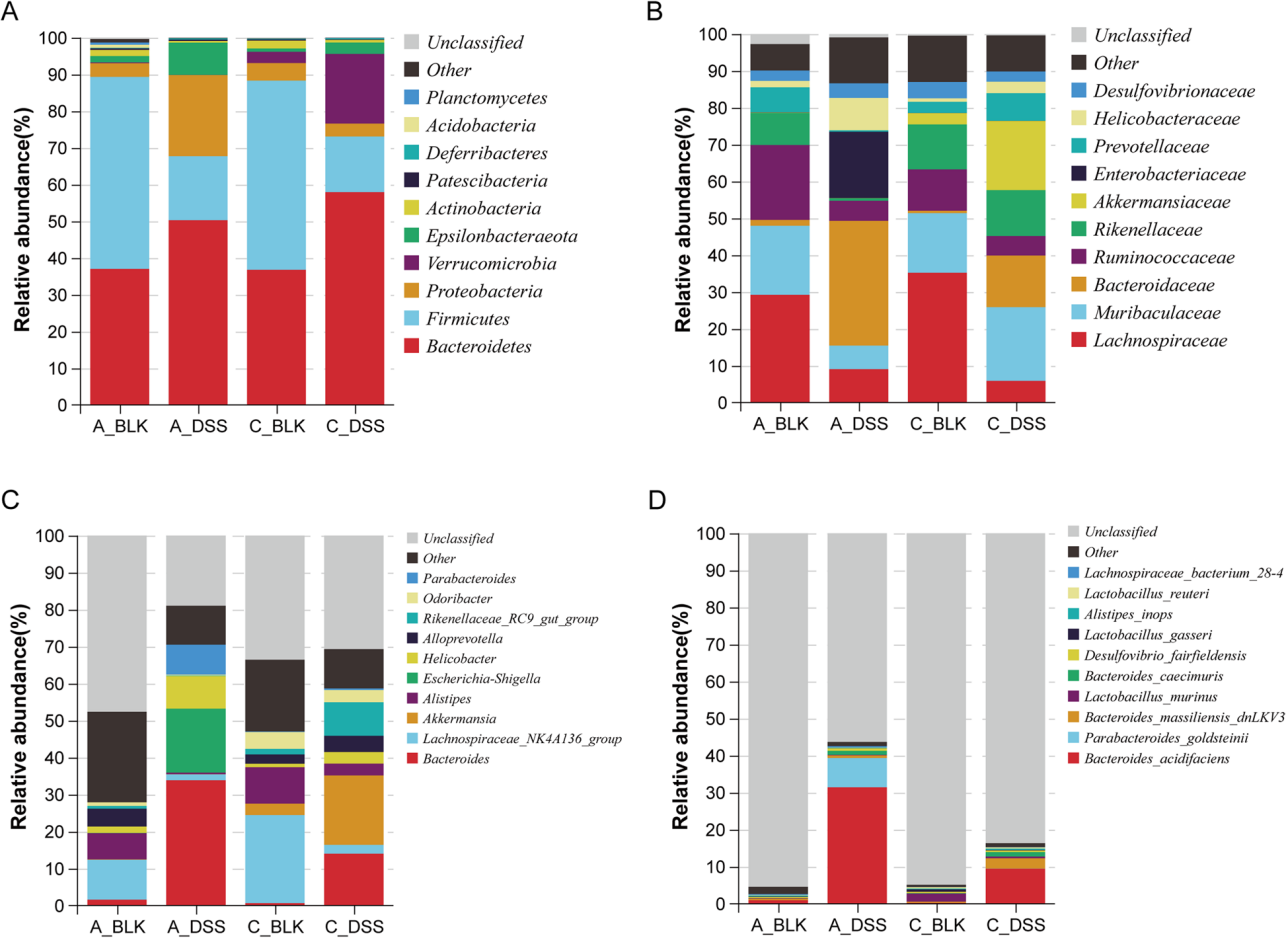
DSS alters dominant gut microbiota structures across different levels in mice with colitis. Stacked bar plot depicts the phylum (**A**) / family (**B**) / genus (**C**) / species (**D**) structure of gut microbiota in each group of mice

As shown in Fig. 3A**,** the relative abundance of *Bacteroidetes* (50.28% vs. 37.05%, *P* = 0.037), *Proteobacteria* (22.09% vs. 3.73%, *P* = 0.006), *Epsilonbacteraeota* (8.79% vs. 1.74%, *P* = 0.037) and *Deferribacteres* (0.31% vs. 0.15%, *P* = 0.337) were notably elevated in mice with acute colitis in comparison to their controls, while that of *Firmicutes* (17.44% vs. 52.23%, *P* = 0.004), *Verrucomicrobia* (0.03% vs. 0.22%, *P* = 0.004), *Actinobacteria* (0.45% vs. 1.66%, *P* = 0.055), *Patescibacteria* (0.4% vs. 0.44%, *P* = 0.631), *Acidobacteria* (0.0005% vs. 0.82%, *P* = 0.003) and *Planctomycetes* (0% vs. 0.64%, *P* = 0.002) were decreased. Likewise, chronic colitis was linked to a significant increase in the relative proportions of *Bacteroidetes* (57.95% vs. 36.77%, *P* = 0.004), *Verrucomicrobia* (18.99% vs. 3.07%, *P* = 0.010), *Epsilonbacteraeota* (3.12% vs. 0.93%, *P* = 0.006), *Deferribacteres* (0.27% vs. 0.15%, *P* = 0.078), *Acidobacteria* (0.02% vs. 0.01%, *P* = 0.935) and *Planctomycetes* (0.08% vs. 0.01%, *P* = 0.150), and a decrease in that of *Firmicutes* (15.12% vs. 51.51%, *P* = 0.004), *Proteobacteria* (3.50% vs. 4.76%, *P* = 0.423), *Actinobacteria* (0.67% vs. 2.11%, *P* = 0.025) and *Patescibacteria* (0.17% vs. 0.35%, *P* = 0.150) compared to the corresponding control.

At the family level (Fig. 3B), *Bacteroidaceae* (33.85% vs. 1.57%, *P* = 0.004), *Enterobacteriaceae* (18.03% vs. 0.09%, *P* = 0.004), *Helicobacteraceae* (8.79% vs. 1.74%, *P* = 0.037) and *Desulfovibrionaceae* (3.94% vs. 2.81%, *P* = 0.749) showed increased abundance in the acute colitis group compared to the control, and *Lachnospiraceae* (9.04% vs. 29.22%, *P* = 0.004), *Muribaculaceae* (6.39% vs. 18.74%, *P* = 0.004), *Ruminococcaceae* (5.44% vs. 20.33%, *P* = 0.004), *Rikenellaceae* (0.69% vs. 8.69%, *P* = 0.004), *Akkermansiaceae* (0.03% vs. 0.10%, *P* = 0.037) and *Prevotellaceae* (0.38% vs. 6.77%, *P* = 0.004) had lower abundance. Furthermore, chronic colitis was associated with a higher abundance of *Muribaculaceae* (19.99% vs. 16.15%, *P* = 0.078), *Bacteroidaceae* (14.01% vs. 0.65%, *P* = 0.004), *Rikenellaceae* (12.46% vs. 12.22%, *P* = 0.262), *Akkermansiaceae* (18.78% vs. 3.07%, *P* = 0.010), *Prevotellaceae* (7.49% vs. 3.02%, *P* = 0.025) and *Helicobacteraceae* (3.12% vs. 0.93%, *P* = 0.006), and lower abundance of *Lachnospiraceae* (5.88% vs. 35.21%, *P* = 0.004), *Ruminococcaceae* (5.28% vs. 11.21%, *P* = 0.004), *Enterobacteriaceae* (0.03% vs. 0.04%, *P* = 0.337) and *Desulfovibrionaceae* (2.76% vs. 4.46%, *P* = 0.200).

Microbiome genera which displayed notable elevations in the acute colitis group compared to the control (Fig. 3C) included *Bacteroides* (33.85% vs. 1.57%, *P* = 0.004), *Escherichia-Shigella* (17.30% vs. 0.06%, *P* = 0.004), *Helicobacter* (8.79% vs. 1.74%, *P* = 0.037) and *Parabacteroides* (8.09% vs. 0.02%, *P* = 0.004), while the abundance of *Lachnospiraceae_NK4A136_group* (1.6% vs. 10.79%, *P* = 0.004), *Akkermansia* (0.03% vs. 0.1%, *P* = 0.037), *Alistipes* (0.42% vs. 7.1%, *P* = 0.004), *Alloprevotella* (0% vs. 4.8%, *P* = 0.002), *Rikenellaceae_RC9_gut_group* (0.15% vs. 0.77%, *P* = 0.016) and *Odoribacter* (0.24% vs. 0.94%, *P* = 0.016) was lower in the acute colitis cohort. The relative abundance of *Bacteroides* (14.01% vs. 0.65%, *P* = 0.004), *Akkermansia* (18.78% vs. 3.07%, *P* = 0.010), *Escherichia-Shigella* (0.02% vs. 0.005%, *P* = 0.871), *Helicobacter* (3.12% vs. 0.93%, *P* = 0.006), *Alloprevotella* (4.39% vs. 2.52%, *P* = 0.262), *Rikenellaceae_RC9_gut_group* (9.06% vs. 1.52%, *P* = 0.004) and *Parabacteroides* (0.42% vs. 0.11%, *P* = 0.016) was higher in the chronic colitis group, while that of *Lachnospiraceae_NK4A136_group* (2.37% vs. 23.80%, *P* = 0.004), *Alistipes* (3.18% vs. 9.86%, *P* = 0.004) and *Odoribacter* (3.31% vs. 4.53%, *P* = 0.262) was lower.

At the species level (Fig. 3D), acute colitis increased the relative abundance of *Bacteroides_acidifaciens* (31.41% vs. 0.94%, *P* = 0.004), *Parabacteroides_goldsteinii* (7.91% vs. 0.005%, *P* = 0.004), *Lactobacillus_murinus* (0.14% vs. 0.12%, *P* = 1.000), *Bacteroides_massiliensis_dnLKV3* (0.72% vs. 0.44%, *P* = 0.337), *Bacteroides_caecimuris* (1.09% vs. 0.04%, *P* = 0.004), *Desulfovibrio_fairfieldensis* (0.68% vs. 0.28%, *P* = 0.200) and *Lachnospiraceae_bacterium_28–4* (0.33% vs. 0.25%, *P* = 0.423), and decreased that of *Lactobacillus_gasseri* (0.03% vs. 0.12%, *P* = 0.022), *Alistipes_inops* (0.14% vs. 0.24%, *P* = 0.150) and *Lactobacillus_reuteri* (0.08% vs. 0.16%, *P* = 0.150). The relative abundance of *Bacteroides_acidifaciens* (9.44% vs. 0.21%, *P* = 0.004), *Parabacteroides_goldsteinii* (0.03% vs. 0.002%, *P* = 0.031), *Bacteroides_massiliensis_dnLKV3* (2.78% vs. 0.34%, *P* = 0.004), *Bacteroides_caecimuris* (1.23% vs. 0.08%, *P* = 0.004), *Desulfovibrio_fairfieldensis* (0.41% vs. 0.38%, *P* = 1.000), *Alistipes_inops* (0.36% vs. 0.22%, *P* = 0.150) and *Lachnospiraceae_bacterium_28–4* (0.05% vs. 0.008%, *P* = 0.004) was higher in the chronic colitis group, while that of *Lactobacillus_murinus* (0.47% vs. 2.2%, *P* = 0.522), *Lactobacillus_gasseri* (0.14% vs. 0.68%, *P* = 0.037) and *Lactobacillus_reuteri* (0.34% vs. 0.36%, *P* = 1.000) was lower compared to the control group.

### Acute and chronic DSS-induced colitis led to distinct changes in the intestinal microbiota

**Fig. 4:**
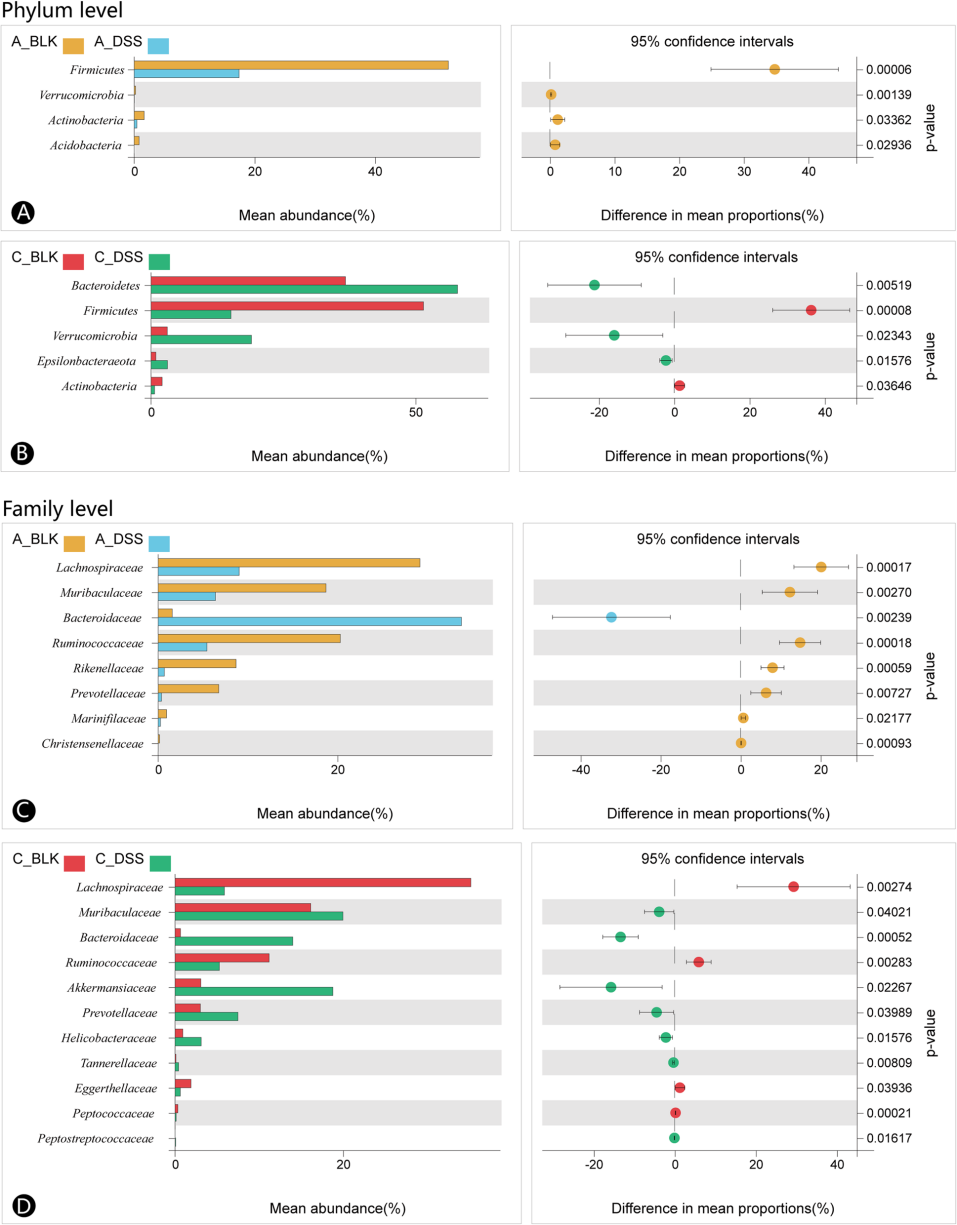
DSS alters gut microbiota structure in acute and chronic colitis mice differently at the phylum and family levels. **A** DSS alters the microbial structure of acute colitis mice at the phylum level. **B** DSS alters the microbial structure of chronic colitis mice at the phylum level. **C** DSS alters the microbial structure of acute colitis mice at the family level. **D** DSS alters the microbial structure of chronic colitis mice at the family level

Welch’s t test was used to analyze the microbial changes induced by acute colitis and chronic colitis. There was a lower abundance of the phyla *Firmicutes*, *Verrucomicrobia*, *Actinobacteria*, *Acidobacteria* and *Planctomycetes* in the acute colitis group compared to the control (Fig. 4A and B). On the other hand, the chronic colitis group, demonstrated increased abundance of *Bacteroidetes*, *Verrucomicrobia* and *Epsilonbacteraeota*, and decreased proportions of *Firmicutes* and *Actinobacteria*. As shown in Fig. 4C and D, the relative proportion of the *Bacteroidaceae* family increased remarkably in the acute colitis group, and that of *Lachnospiraceae*, *Muribaculaceae*, *Ruminococcaceae*, *Rikenellaceae*, *Prevotellaceae*, *Marinifilaceae* and *Christensenellaceae* decreased compared to the control. Furthermore, *Muribaculaceae*, *Bacteroidaceae*, *Akkermansiaceae*, *Prevotellaceae, Helicobacteraceae*, *Tannerellaceae* and *Peptostreptococcaceae* were highly abundant in the chronic colitis group relative to the control, while *Lachnospiraceae*, *Ruminococcaceae*, *Eggerthellaceae* and *Peptococcaceae* showed low abundance.

**Fig. 5:**
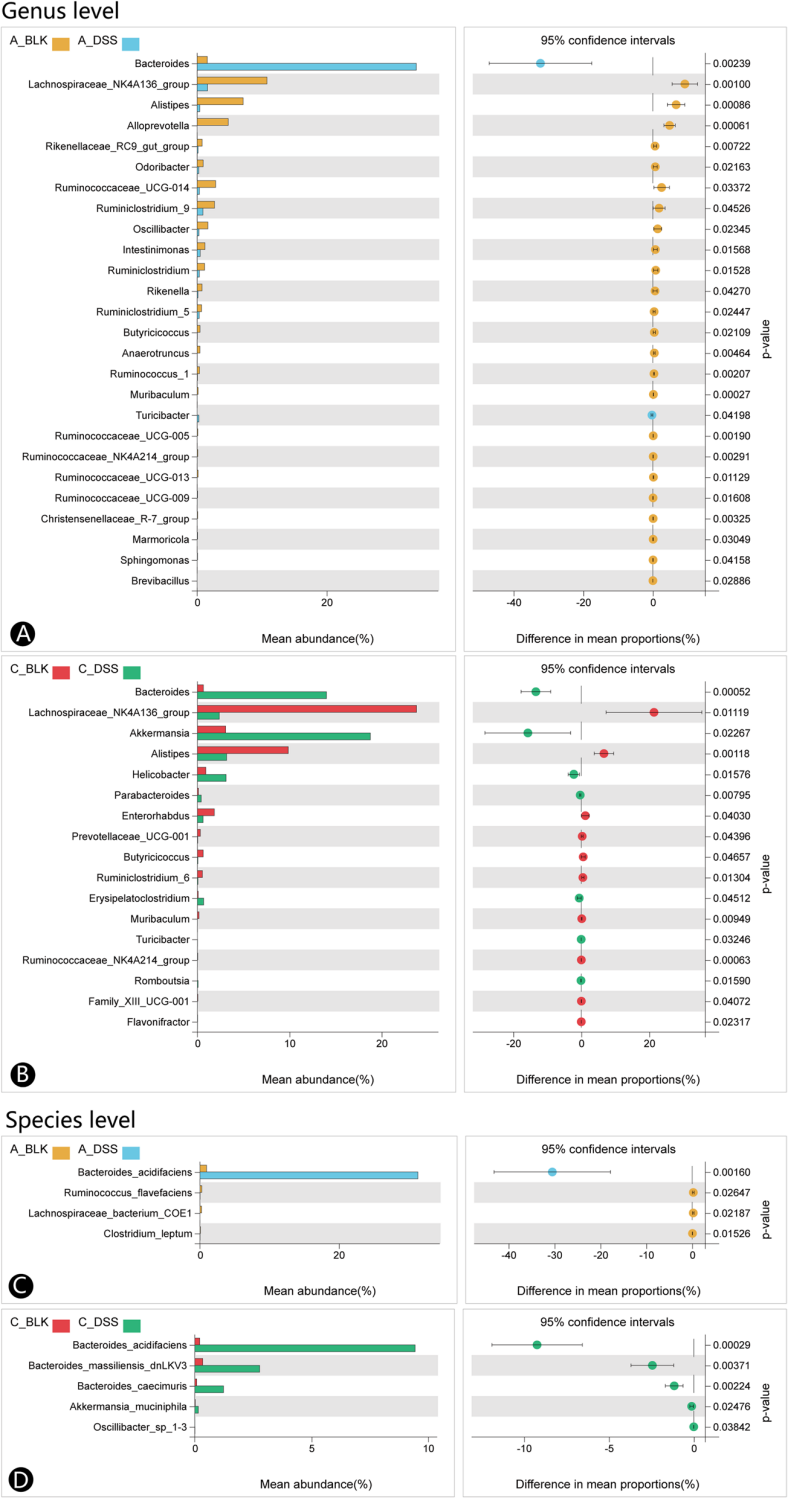
DSS alters gut microbiota structure in acute and chronic colitis mice differently at the genus and species levels. **A** DSS alters the microbial structure of acute colitis mice at the genus level. **B** DSS alters the microbial structure of chronic colitis mice at the genus level. **C** DSS alters the microbial structure of acute colitis mice at the species level. **D** DSS alters the microbial structure of chronic colitis mice at the species level

As shown in Fig. 5A and B, the relative abundance of the *Bacteroides* and *Turicibacter* families was increased, while that of *Lachnospiraceae_NK4A136_group*, *Alistipes*, *Alloprevotella*, *Rikenellaceae_RC9_gut_group*, *Odoribacter*, *Ruminococcaceae_UCG-014*, *Ruminiclostridium_9*, *Oscillibacter*, *Intestinimonas*, *Ruminiclostridium*, *Rikenella*, *Ruminiclostridium_5*, *Butyricicoccus*, *Anaerotruncus*, *Ruminococcus_1*, *Muribaculum*, *Ruminococcaceae_UCG-005*, *Christensenellaceae_R-7_group*, *Ruminococcaceae_NK4A214_group*, *Ruminococcaceae_UCG-009*, *Ruminococcaceae_UCG-013*, *Marmoricola*, *Sphingomonas* and *Brevibacillus* decreased significantly in the acute colitis group. In the chronic colitis group, the most abundant genera relative to the control group were *Bacteroides*, *Akkermansia*, *Helicobacter*, *Parabacteroides*, *Erysipelatoclostridium*, *Turicibacter* and *Romboutsia*, whereas *Lachnospiraceae_NK4A136_group*, *Alistipes*, *Enterorhabdus*, *Prevotellaceae_UCG-001*, *Butyricicoccus*, *Ruminiclostridium_6*, *Muribaculum*, *Ruminococcaceae_NK4A214_group*, *Family_XIII_UCG-001* and *Flavonifractor* had low abundance. At the species level (Fig. 5C and D), acute colitis drastically elevated the relative proportion of *Bacteroides_acidifaciens*, and lowered that of *Ruminococcus_flavefaciens*, *Lachnospiraceae_bacterium_COE1* and *Clostridium_leptum*. Chronic colitis increased the abundance of *Bacteroides_acidifaciens*, *Bacteroides_massiliensis_dnLKV3*, *Bacteroides_caecimuris*, *Akkermansia_muciniphila* and *Oscillibacter_sp_1–3*. Thus, the proportion of SCFAs-producing bacteria was elevated in the intestines of younger mice exposed to DSS over a longer duration. In contrast, acute colitis increased the abundance of conditional pathogens.

## Discussion

DSS and 2,4,6-trinitro-benzenesulfonic acid (TNBS) are established agents for inducing acute and chronic colitis [17–20]. In this preliminary study, we found that administration of 3 and 2.5% DSS via drinking water can respectively induce acute and chronic colitis in mice while ensuring their survival. The affected mice showed significantly increased DAI scores, shortened colons, and significant damage to the intestinal mucosa. Interestingly, the weight of the control mice also fluctuated during the 5-week long cycle of chronic colitis modeling, which resulted in a transient increase in DAI score. However, the control group of the acute model was not affected due to the short treatment duration. Likewise, the colonic tissue of older mice also displayed limited neutrophil infiltration that did not penetrate beyond the lamina propria. In addition, the alternating active and remission phases during the induction of chronic colitis led to localized thickening of colonic mucosa, in contrast to the uniform thinning observed in acute colitis. Our models of acute and chronic colitis demonstrated good stability and homogeneity with a lack of significant variability in terms of disease features.

However, DSS-induced acute colitis led to notable reduction in the α-diversity of the gut microbiota compared to chronic colitis. Further distinctions between the two groups were highlighted with β-diversity analysis. Table S1 summarizes the changes in microbial structure at the levels of the phylum, family, genus and species. DSS-induced colitis stimulated the growth of *Bacteroidetes*, *Epsilonbacteraeota* and *Deferribacteres*, while lowering that of *Firmicutes*, *Actinobacteria* and *Patescibacteria*. The *Firmicutes*/*Bacteroidetes* ratio in the gut is an indicator of intestinal inflammation, and increases during colitis and other inflammatory conditions [21, 22]. In addition, the increased abundance of some conditional pathogens is also an important intestinal flora feature of UC. Li et al [23]. collected 56 mucosal microbiome samples from 28 Chinese UC patients and their healthy family partners, and identified four opportunistic “pathogens” (i.e., *Clostridium tertium*, *Odoribacter splanchnicus*, *Ruminococcus gnavus* and *Flavonifractor plautii*) with potential diagnostic and therapeutic significance for UC, which were inhibited in healthy individuals. Moreover, Huh et al. [24] identified *Fusobacterium nucleatum* as a marker for early gut microbial dysbiosis in inflammatory bowel diseases.

In our study, DSS-induced acute and chronic colitis simulated UC-associated microbial changes. Acute colitis raised the proportion of the *Proteobacteria* phylum and lowered that of the *Verrucomicrobia* phylum, whereas chronic colitis had the opposite effect. *Proteobacteria* is enriched in UC patients, which correlates with the significant reduction in intestinal SCFAs content (especially in the active phase) [25, 26]. In addition, the *Verrucomicrobia* phylum includes important SCFAs-producing bacteria such as *Ruminococcus* and *Akkermansia*. The abundance of the *Bacteroidaceae* and *Helicobacteraceae* families had increased, while that of *Lachnospiraceae* and *Ruminococcaceae* decreased in mice with acute and chronic colitis. The *Helicobacteraceae* family consists of conditional pathogens [27, 28], while *Lachnospiraceae* and *Ruminococcaceae* include SCFAs-producing bacteria that are negatively correlated with colitis [29–31]. *Enterobacteriaceae* and *Desulfovibrionaceae* were abundant in mice with acute colitis, while *Rikenellaceae*, *Akkermansiaceae* and *Prevotellaceae* showed lower abundance. Excessive proliferation of *Enterobacteriaceae* has been observed in the gut of UC patients [32, 33]. Surprisingly, the SCFAs-producing *Rikenellaceae*, *Akkermansiaceae* and *Prevotellaceae* were raised in mice with chronic colitis, which is contradictory to existing reports. Furthermore, both acute and chronic colitis were associated with a higher abundance of opportunistic pathogens (*Bacteroides*, *Escherichia-Shigella*, *Helicobacter*, *Bacteroides_acidifaciens*, *Bacteroides_massiliensis_dnLKV3* and *Bacteroides_caecimuris*), and lower abundance of probiotics/SCFAs-producing bacteria (*Lachnospiraceae_NK4A136_group*, *Alistipes*, *Butyricicoccus*, *Ruminococcaceae_NK4A214_group*, *Lactobacillus_gasseri* and *Lactobacillus_reuteri*). Interestingly, the inhibition of SCFAs-producing bacteria was significantly stronger in mice with acute colitis compared to the chronic colitis model, especially for *Rikenellaceae*, *Ruminococcaceae*, *Ruminiclostridium*, *Alistipes* and *Clostridium_leptum*. These differences can be attributed to the longer modeling duration of chronic colitis as well as the higher concentration of DSS used to simulate acute colitis. High levels of DSS decreased food intake and resulted in more severe diarrhea and bloody stool symptoms, which lowered the impact of diet and fecal preference on the gut microbiota. Furthermore, the local thickening of the colonic mucosa during chronic colitis likely increased the proliferation of *Akkermansia_Muciniphila*, a myxotrophin bacterium related to mucous production and intestinal metabolism [34]. This organism is linked to a number of chronic conditions including diabetes and non-alcoholic steatohepatitis, and may be responsible for the aberrant mucin secretion during chronic colitis. Finally, *Rikenellaceae*, *Ruminococcaceae*, *Ruminiclostridium*, *Alistipes* and *Clostridium_leptum* thrive in an anaerobic environment, which is possibly disrupted during the induction of acute colitis, resulting in lower proliferation of these bacteria.

However, several limitations of the present study should be noted. First, the analysis of the acute colitis models can be performed within 1–2 weeks, whereas that of the chronic models may take 2–4 months [9]. In the present study, in order to ensure the survival rate of mice and effects of prolonged feeding on gut microbiota, we modeled colitis over a shorter period. Second, we did not assess the impact of different DSS concentrations and administration methods (such as oral gavage) on the intestinal microbiota. Third, we did not compare the effects of acute/chronic DSS exposure in different mouse strains (such as C57BL/6). Finally, the microbiota research method used in this study is relatively simple, and network analysis [23] of differential OTUs may adapt to reveal the complex relationships among microbes. Unfortunately, due to the small sample size of this study and our limited technical expertise in bioinformatics, we did not perform network analysis. However, these issues will be addressed in our subsequent study.

To summarize, both acute and chronic colitis stimulated changes in the gut microbiota, and the alterations in the acute colitis model are more consistent with that observed in UC patients. Furthermore, acute colitis inhibited the SCFAs-producing bacteria to a significantly greater extent compared to chronic colitis. Finally, younger age and shorter exposure to DSS are more conducive to the stability of the colitis microbiota in the mouse model. Our study provides an experimental basis for the analysis of colitis microbiota, and clearly shows that the acute colitis model simulates UC to a greater extent compared to the chronic colitis model.

## Conclusions

DSS-induced acute and chronic colitis demonstrated similar symptoms and histopathological changes. However, the alterations in the acute colitis model are more consistent with that observed in UC patients than chronic colitis models. Acute colitis induced higher abundance of SCFAs-producing bacteria and lower α-diversity compared to chronic colitis.

## Materials and methods

### Establishment of DSS-induced colitis and treatment

This study was carried out in compliance with the ARRIVE guidelines. All animal experiment protocols were approved by the Animal Ethics Committee of Guangzhou First People’s Hospital (Approval number: 2017–202). Male BALB/c mice (*n* = 24, aged 6–8 weeks) were purchased from Guangdong Medical Laboratory Animal Center (Foshan, China, Certificate number SYXK 2013–0002). Animals were reared in a specific pathogen-free (SPF) environment at 24 °C under a 12-h light/dark cycle and relative humidity 50–70%. Mice were allowed free access to food and water. The mice were randomly divided into the acute blank (A_BLK), acute DSS-induced colitis (A_DSS), chronic blank (C_BLK) and chronic DSS-induced colitis (C_DSS) groups (*n* = 6 each). The control groups received regular drinking water for 1 week and 5 weeks respectively. Mice were stimulated to develop acute colitis through the addition of 3% DSS (MP Biomedicals, USA) into their drinking water for a week. Chronic colitis was induced by supplementing their drinking water with 2.5% DSS every other week for 5 weeks [8, 35]. Daily assessments of mice body weights, stool consistency, as well as the presence of blood-stained stool and anus were carried out. Mice were anesthetized with pentobarbital sodium and euthanized via transcardiac perfusion at the end of the experiments, and their colons were harvested, measured, and fixed overnight in 10% neutral buffered formalin. Stool samples were also collected from the cecum.

### DAI scoring

A previously published protocol was adapted to assess the Disease Activity Index (DAI) [36] based on the following parameters: 1) weight loss, in terms of percentage from original body weight (0 – no loss; 1–1–5%; 2–5–10%; 3–10–15%; and 4 - > 15%), 2) stool consistency (0 - normal; 1 - pasty and not adherent to the anus; 2 - pasty and mildly adherent to the anus; 3 - pasty and adherent to the anus; and 4 - watery), and 3) rectal bleeding (0 - hemoccult (−); 1 - hemoccult (±); 2 - hemoccult (+); 3 - hemoccult (++); and 4 - obvious blood in stool) [37].

### Histopathological evaluation

The entire length of the colon was dissected along the mesenteric border from each mouse, sliced longitudinally, and rinsed with cold saline. The tissue samples were fixed in buffered formalin, embedded in paraffin, and cut into 5 μm-thick sections. The slides were then stained with hematoxylin and eosin (HE) and scored independently by two investigators who were blinded to the grouping as follows: 1) inflammatory changes (0 - no inflammatory cells infiltrate; 1 – inflammatory infiltrates until the lamina propria; 2 – inflammatory infiltrates until the submucosa; and 3 - transmural infiltration), 2) presence of ulceration (0 - no ulceration; 1–1 ~ 2 ulcers; 2–3 ~ 4 ulcers; 3 - > 4 ulcers), 3) mucosal hyperplasia (0 - normal; 1 – mildly fibrosed and thickened mucosa; 2 – fibrous hyperplasia and mucosal thickening; 3 – extensive fibrous hyperplasia or granulation and mucosal thickening), and 4) edema (0 - none; 1–0 ~ 30%; 2–30 ~ 70%; 3 - > 70%) [38]..

### Extraction of fecal DNA and analysis of gut microbiota

The HiPure Stool DNA Kit (Magen, Guangzhou, China) was used to extract total fecal DNA based on instructions provided by the manufacturer. The following primers were used to amplify the *16S rDNA* target regions: 341F: CCTACGGGNGGCWGCAG; 806R: GGACTACHVGGGTATCTAAT [39]. Amplified products of 400–450 bpm were purified using the Phusion High-Fidelity PCR Master Mix (New England Biolabs, Beverly, MA, USA). Sequencing libraries were generated using the TruSeq DNA PCR-Free Sample Preparation Kit (Illumina, San Diego, CA, USA) based on protocols stipulated by the manufacturer before adding in index codes. The quality of the constructed library was evaluated using the Agilent Bioanalyzer 2100 system and Qubit@ 2.0 Fluorometer (Thermo Scientific, Carlsbad, CA, USA), an the Illumina HiSeq 2500 platform (Tianjin Novogene Bioinformatics Technology Co., Ltd) was used for sequencing [39].

### Bioinformatics analyses

#### Quality control and reads assembly

##### Reads filtering

Raw data containing adapters or low-quality reads were further filtered according to the FASTP (version 0.18.0) criteria: 1) containing more than 10% of unknown nucleotides (N) and 2) containing less than 50% of bases with quality (Q-value)>20.

##### Reads assembly

Paired end clean reads were merged as raw tags using FLSAH (version 1.2.11) with a minimum overlap of 10 bp and mismatch error rates of 2%.

##### Raw tag filtering

Noisy sequences of raw tags were filtered by QIIME (version 1.9.1) pipeline under specific filtering conditions [40] to obtain the high-quality clean tags. The filtering conditions are as follows: 1) Break raw tags from the first low quality base site where the number of bases in the continuous low-quality value (the default quality threshold is <=3) reaches the set length (the default length is 3), and 2) filter tags with high-quality base length is less than 75% of the tag length.

In total, we obtained 1,344,698 tags without primers, with 56,029 tags per sample on average. [Link for the NCBI dataset: http://www.ncbi.nlm.nih.gov/bioproject/738354].

### α-Diversity analysis

QIIME (version 1.9.1) was used to derive the α-diversity indices [41]. The ggplot2 package of the R project (version 2.2.1) was used to perform OTU rarefaction and plot rank abundance curves. Various α-indices (Pielou index and Shannon index) between two groups were calculated using the Wilcoxon rank test in the R project Vegan package (version 2.5.3). The Tukey’s HSD test was used to evaluate α-indices (Pielou index and Shannon index) among three or more groups.

### Operational Taxonomic Units (OTUs) analysis and β-diversity analysis

Effective tags of OTUs ≥ 97% were clustered together with using UPARSE software (version 9.2.64) [42]. Each cluster was represented by the most abundant tag sequence of each cluster. The VennDiagram package [43] of the R project (version 1.6.16) was used to analyze element distribution, while the Vegan package in the R project (version 2.5.3) was utilized for principal component analysis (PCA). Relevant sequences were aligned with the software Muscle [44] (version 3.8.31) and clustering trees were constructed using the FastTree [45] (version 2.1) and UPGMA (Unweighted Pair Group Method with Arithmetic Mean) software. Multivariate statistical techniques including PCoA (principal coordinates analysis) [46] and NMDS (non-metric multi-dimensional scaling) [47] of Bray-Curtis distances were calculated using the Vegan package (version 2.5.3) and plotted using ggplot2 package (version 2.2.1).

### Community composition analysis

A naive Bayesian model using RDP classifier [48] (version 2.2) based on SILVA [49] (version 132) or Greengene [50] (version gg_13_5) databases was used to classify sequences based on organisms. The community composition was visualized as a stacked bar plot using ggplot2 package [http://CRAN.R-project.org/package=ggplot2] (version 2.2.1) in the R project.

### Welch’s t test

Welch’s t test was used to evaluate significant variances in terms of mean abundance of species between two groups. When there are two groups in the comparison group and total repeat samples in each group ≥3, the number of species tags/total tags of at least one sample ≥ 0.1%, and the top 1000 high abundance OTUs are statistically tested with R language vegan data package.

### Statistical analysis

Data is depicted in terms of percentage or the mean ± standard deviation (SD). Inter- and intra-group differences were analyzed by the Wilcoxon signed rank test or unpaired t-test, one-way ANOVA and post hoc Tukey’s test as appropriate. The SPSS software (version 23.0; IBM Corp.) was utilized for all statistical analyses. Statistical significance was granted when the *p* values were < 0.05.

## Supplementary Information

**Additional file 1: Figure S1.** DSS alters β-diversity (PCA & PCoA) of gut microbiota in mice models of colitis. (A) PCA (Principal Component Analysis). The PC1 coordinate indicates the primary principal component while its contribution value to sample variability is depicted in percentage in brackets. The PC2 coordinate represents the second principal component while its contribution value to sample variability is depicted in percentage in brackets. Samples are represented by colored dots in the panel. Sample compositions which are similar are located more closely together on the PCA diagram. Samples of various different environments signify their respective aggregation distribution. (B) PCoA (Principal Co-ordinate Analysis). PCoA is a dimension reduction analysis based on the distance matrix, and the percentage (the number in brackets of the axis title) is used to evaluate the explanation degree of each coordinate axis to the overall difference of microbial structure. Generally, it is better that the sum of PCoA1 and PCoA2 is more than 50%. **Table S1.** Alterations of microbial structure at different levels in acute / chronic colitis mice.

## Data Availability

The original contributions presented in the study are publicly available. These data can be found here: http://www.ncbi.nlm.nih.gov/bioproject/738354, BioProject ID is PRJNA 738354. Other datasets used and analyzed during the current study are available from the corresponding authors on reasonable request.

## Abbreviations

DSS: Dextran sulfate sodium
UC: Ulcerative colitis
DAI: Disease activity index
SCFAs: Short chain fatty acids
IBD: Inflammatory bowel disease
CD: Crohn’s disease
OTUs: Operational taxonomic unit
UPGMA: Unweighted pair group method with arithmetic mean
NMDS: Non-metric multi-dimensional scaling
PCA: Principal component analysis
PCoA: Principal coordinates analysis
