# Supplementary material for: Selection strategy of dextran sulfate sodium-induced acute or chronic colitis mouse models based on gut microbial profile
Source: BMC Microbiol. 2021 Oct 16;21:279. doi: 10.1186/s12866-021-02342-8 (PMC8520286; doi:10.1186/s12866-021-02342-8)
Supplement: Supplementary file 1 — Additional file 1: Figure S1. DSS alters β-diversity (PCA & PCoA) of gut microbiota in mice models of colitis. (A) PCA (Principal Component Analysis). The PC1 coordinate indicates the primary principal component while its contribution value to sample variability is depicted in percentage in brackets. The PC2 coordinate represents the second principal component while its contribution value to sample variability is depicted in percentage in brackets. Samples are represented by colored dots in the panel. Sample compositions which are similar are located more closely together on the PCA diagram. Samples of various different environments signify their respective aggregation distribution. (B) PCoA (Principal Co-ordinate Analysis). PCoA is a dimension reduction analysis based on the distance matrix, and the percentage (the number in brackets of the axis title) is used to evaluate the explanation degree of each coordinate axis to the overall difference of microbial structure. Generally, it is better that the sum of PCoA1 and PCoA2 is more than 50%. Table S1. Alterations of microbial structure at different levels in acute / chronic colitis mice. [file 12866_2021_2342_MOESM1_ESM.docx]

**Supplementary Figure and Table:**

**
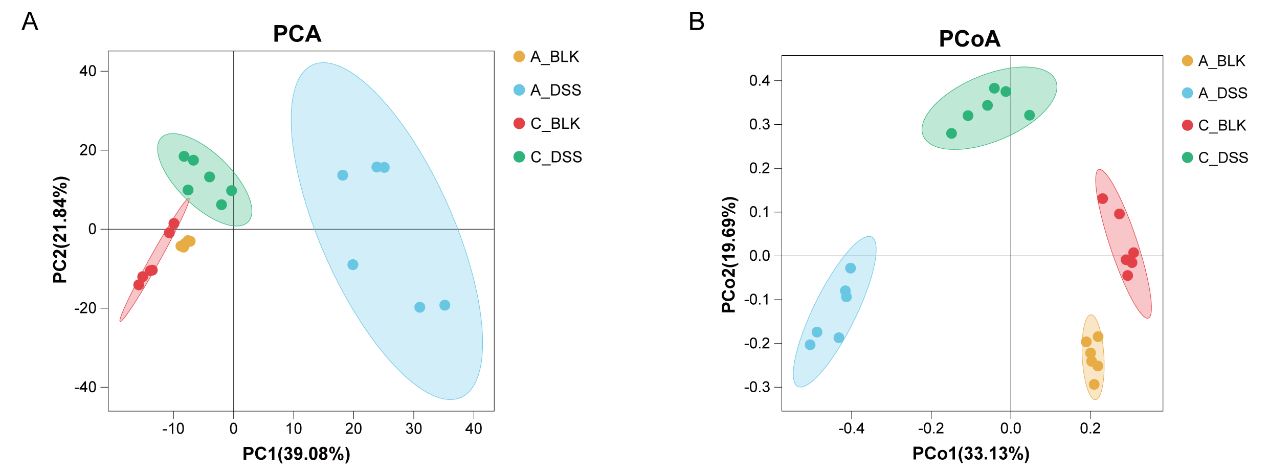
**

**Figure S1. DSS alters β-diversity (PCA & PCoA) of gut microbiota in mice models of colitis.**

(A) PCA (Principal Component Analysis). The PC1 coordinate indicates the primary principal component while its contribution value to sample variability is depicted in percentage in brackets. The PC2 coordinate represents the second principal component while its contribution value to sample variability is depicted in percentage in brackets. Samples are represented by colored dots in the panel. Sample compositions which are similar are located more closely together on the PCA diagram. Samples of various different environments signify their respective aggregation distribution. (B) PCoA (Principal Co-ordinate Analysis). PCoA is a dimension reduction analysis based on the distance matrix, and the percentage (the number in brackets of the axis title) is used to evaluate the explanation degree of each coordinate axis to the overall difference of microbial structure. Generally, it is better that the sum of PCoA1 and PCoA2 is more than 50%.

| **Table S1. Alterations of microbial structure at different levels in acute / chronic colitis mice** | | |
| --- | --- | --- |
|  | **Increase** | **Decrease** |
| **Phylum** | *Bacteroidetes*(both) | *Firmicutes*(both) |
|  | *Epsilonbacteraeota*(both) | *Actinobacteria*(both) |
|  | *Deferribacteres*(both) | *Patescibacteria*(both) |
|  | *Proteobacteria*(acute) | *Verrucomicrobia*(acute) |
|  | *Verrucomicrobia*(chronic) | *Acidobacteria*(acute) |
|  | *Acidobacteria*(chronic) | *Planctomycetes*(acute) |
|  | *Planctomycetes*(chronic) | *Proteobacteria*(chronic) |
|  |  |  |
| **Family** | *Bacteroidaceae*(both) | *Lachnospiraceae*(both) |
|  | *Helicobacteraceae*(both) | *Ruminococcaceae*(both) |
|  | *Enterobacteriaceae*(acute) | *Muribaculaceae*(acute) |
|  | *Desulfovibrionaceae*(acute) | *Rikenellaceae*(acute) |
|  | *Muribaculaceae*(chronic) | *Akkermansiaceae*(acute) |
|  | *Rikenellaceae*(chronic) | *Prevotellaceae*(acute) |
|  | *Akkermansiaceae*(chronic) | *Marinifilaceae*(acute) |
|  | *Prevotellaceae*(chronic) | *Christensenellaceae*(acute) |
|  | *Tannerellaceae*(chronic) | *Enterobacteriaceae*(chronic) |
|  | *Peptostreptococcaceae*(chronic) | *Desulfovibrionaceae*(chronic) |
|  |  | *Eggerthellaceae*(chronic) |
|  |  | *Peptococcaceae*(chronic) |
|  |  |  |
| **Genus** | *Bacteroides*(both) | *Lachnospiraceae_NK4A136_group*(both) |
|  | *Escherichia-Shigella*(both) | *Alistipes*(both) |
|  | *Helicobacter*(both) | *Odoribacter*(both) |
|  | *Parabacteroides*(both) | *Butyricicoccus*(both) |
|  | *Turicibacter*(acute) | *Muribaculum*(both) |
|  | *Akkermansia*(chronic) | *Ruminococcaceae_NK4A214_group*(both) |
|  | *Alloprevotella*(chronic) | *Akkermansia*(acute) |
|  | *Rikenellaceae_RC9_gut_group*(chronic) | *Alloprevotella*(acute) |
|  | *Erysipelatoclostridium*(chronic) | *Rikenellaceae_RC9_gut_group*(acute) |
|  | *Turicibacter*(chronic) | *Ruminococcaceae_UCG-014*(acute) |
|  | *Romboutsia*(chronic) | *Ruminiclostridium_9*(acute) |
|  |  | *Intestinimonas*(acute) |
|  |  | *Ruminiclostridium*(acute) |
|  |  | *Rikenella*(acute) |
|  |  | *Ruminiclostridium_5*(acute) |
|  |  | *Anaerotruncus*(acute) |
|  |  | *Ruminococcus_1*(acute) |
|  |  | *Ruminococcaceae_UCG-005*(acute) |
|  |  | *Ruminococcaceae_UCG-013*(acute) |
|  |  | *Ruminococcaceae_UCG-009*(acute) |
|  |  | *Christensenellaceae_R-7_group*(acute) |
|  |  | *Marmoricola*(acute) |
|  |  | *Sphingomonas*(acute) |
|  |  | *Brevibacillus*(acute) |
|  |  | *Enterorhabdus*(chronic) |
|  |  | *Prevotellaceae_UCG-001*(chronic) |
|  |  | *Ruminiclostridium_6*(chronic) |
|  |  | *Family_XIII_UCG-001*(chronic) |
|  |  | *Flavonifractor*(chronic) |
|  |  |  |
| **Species** | *Bacteroides_acidifaciens*(both) | *Lactobacillus_gasseri*(both) |
|  | *Parabacteroides_goldsteinii*(both) | *Lactobacillus_reuteri*(both) |
|  | *Bacteroides_massiliensis_dnLKV3*(both) | *Alistipes_inops*(acute) |
|  | *Bacteroides_caecimuris*(both) | *Ruminococcus_flavefaciens*(acute) |
|  | *Desulfovibrio_fairfieldensis*(both) | *Lachnospiraceae_bacterium_COE1*(acute) |
|  | *Lachnospiraceae_bacterium_28-4*(both) | *Clostridium_leptum*(acute) |
|  | *Lactobacillus_murinus*(acute) | *Lactobacillus_murinus*(chronic) |
|  | *Alistipes_inops*(chronic) |  |
|  | *Akkermansia_muciniphila*(chronic) |  |
|  | *Oscillibacter_sp_1-3*(chronic) |  |
